# Hybrid, ultra-deep metagenomic sequencing enables genomic and functional characterization of low-abundance species in the human gut microbiome

**DOI:** 10.1080/19490976.2021.2021790

**Published:** 2022-01-22

**Authors:** Hao Jin, Lijun You, Feiyan Zhao, Shenghui Li, Teng Ma, Lai-Yu Kwok, Haiyan Xu, Zhihong Sun

**Affiliations:** Key Laboratory of Dairy Biotechnology and Engineering, Ministry of Education, Key Laboratory of Dairy Products Processing, Ministry of Agriculture and Rural Affairs, Inner Mongolia Key Laboratory of Dairy Biotechnology and Engineering, Inner Mongolia Agricultural University, Hohhot, China

**Keywords:** Whole-metagenome shotgun sequencing, PacBio SMRT sequencing, low-abundance species, metagenomic-assembled genome (MAG), complete (circularized, no gaps) MAG (CMAG)

## Abstract

A large number of microbial genomes have already been identified from the human gut microbiome, but the understanding of the role of the low-abundance species at the individual level remains challenging, largely due to the relatively shallow sequencing depth used in most studies. To improve genome assembling performance, a HiSeq-PacBio hybrid, ultra-deep metagenomic sequencing approach was used to reconstruct metagenomic-assembled genomes (MAGs) from 12 fecal samples. Such approach combined third-generation sequencing with ultra-deep second-generation sequencing to improve the sequencing coverage of the low-abundance subpopulation in the gut microbiome. Our study generated a total of 44 megabase-scale scaffolds, achieving four single-scaffolds of complete (circularized, no gaps) MAGs (CMAGs) that were the first circular genomes of their species. Moreover, 475 high-quality MAGs were assembled across all samples. Among them, 234 MAGs were currently uncultured, including 24 MAGs that were not found in any public genome database. Additionally, 287 and 77 MAGs were classified as low-abundance (0.1–1%) and extra-low-abundance (<0.1%) gut species in each individual, respectively. Our results also revealed individual-specific genomic features in the MAG profiles, including microbial genome growth rate, selective pressure, and frequency of chromosomal mobile genetic elements. Finally, thousands of extrachromosomal mobile genetic elements were identified from the metagenomic data, including 5097 bacteriophages and 79 novel plasmid genomes. Overall, our strategy represents an important step toward comprehensive genomic and functional characterization of the human gut microbiome at an individual level.

## Introduction

Trillions of microbes colonize the human colon, representing a large reservoir of organisms that co-exist with humans.^1^ In the last two decades, tremendous amount of research has revealed the pivotal roles of the gut microbiota in host health and disease.^2–4^ The presence of a large variety of gut microbes is essential for the assimilation and metabolism of both exogenous and endogenous substrates, and for shaping the host physiology in multiple ways.^5–7^ Under healthy conditions, the normal microbiota is distributed across different niches of the host system based on available nutrients and host defense.^8,9^ Therefore, discovering functional information from complex microbial communities in an individual can enhance the understanding of host-microbe interactions.

However, our understanding of the human gut microbiome has been constrained by the high proportion of uncultured gut colonizing microbes and the lack of a high-quality reference genome. This is particularly true for the low-abundance microbial species.^10^ Metagenomics provides a culture-independent way to explore these unknown species. Recent studies in this field have attempted to reconstruct microbial genomes from metagenomes known as metagenome-assembled genomes (MAGs).^11–13^ The availability of a large number of novel MAGs not only significantly improves raw-read mappability, but helps reveal functional metagenomic potential and possible correlation between metagenome features and human disease.^13–15^ Previous metagenomic sequencing studies have been performed based on large scale samples but were limited to a small data size of approximately 5 ~ 10 Gbp per sample.^11–13^ This sequencing depth is not enough to capture the low-abundance microbial genomes, and much information of each metagenomic sample is therefore lost. Theoretically, a sequence data size of 5 Gbp per sample would only provide a limited genome coverage (i.e., 5 Mbp data) for a species of 0.1% relative abundance in that sample, making it almost impossible for accurate and deep comparative metagenomic analysis. Indeed, some gut microbes that play an incredibly profound role in human health may be present only as low-abundance species. For example, individual members of lactic acid bacteria often comprise less than 0.1% of the human gut microbiota.^16^ Thus, profiling the low-abundance species may provide new insights into the understanding of the gut ecology and function of the gut microbiome.

Long-read sequencing approaches may alleviate many challenges currently faced by short-read sequencing (e.g., Illumina HiSeq) in metagenomic assembly.^17,18^ For example, long-read sequencing can cover repetitive and low-coverage regions, and thus increases assembly contiguity.^19^ Therefore, much interest has been shown in long-read assembly, as it holds great promise for a better understanding of complex metagenomic communities, including microbiomes from the environment, rumen, skin, and human gut.^20–22^ For example, complete (circularized, no gaps) MAGs (CMAGs) have been directly assembled from complex human gut metagenomes by using the nanopore sequencing platform.^23^ Single-molecule, real-time (SMRT) sequencing has been shown to boost performance of single genome assembly,^24^ as well as metagenome assembly for identifying host-plasmid/virus associations in metagenomic analysis.^25–28^ Long-read sequencing has facilitated the development of high-quality genome assembly, but assembling genomes for all microbes within a single individual remains challenging, largely due to the relatively shallow sequencing depth used in most studies. This does not only limit our ability to obtain an exquisitely detailed view of the gut ecology, but also hinders the development of gut microbiome-based personalized medicine. Here, a HiSeq-PacBio hybrid, ultra-deep metagenomic sequencing approach was designed and used to reconstruct MAGs from 12 fecal samples obtained from eight human subjects. To improve assembly performance, the Human Microbiome Project (HMP) mock dataset was used to compare the power of different metagenome assembly approaches. Our results showed that the currently designed strategy exceeded existing methods in genomic and functional characterization of low- and extra-low abundance species in the human gut microbiome.

## Results

### Metagenomic sequencing and assembly strategy

Two whole-metagenome shotgun sequencing datasets were analyzed in-depth to characterize the genomic features of the human gut microbiome in the collected samples. The first dataset included sequences of eight samples from four different individuals (two samples from each individual taken seven days apart). These samples were sequenced on both HiSeq and PacBio platforms (generated a total of 274 Gbp data; 34.2 ± 10.8 Gbp of HiSeq and 8.7 ± 3.7 Gbp of PacBio data per sample, respectively; Table S1a). The second dataset comprised sequencing data from four samples collected from four individuals (a total of 277 Gbp HiSeq data, 69.3 ± 39.8 Gbp per sample; Table S1a).

**Figure 1.**
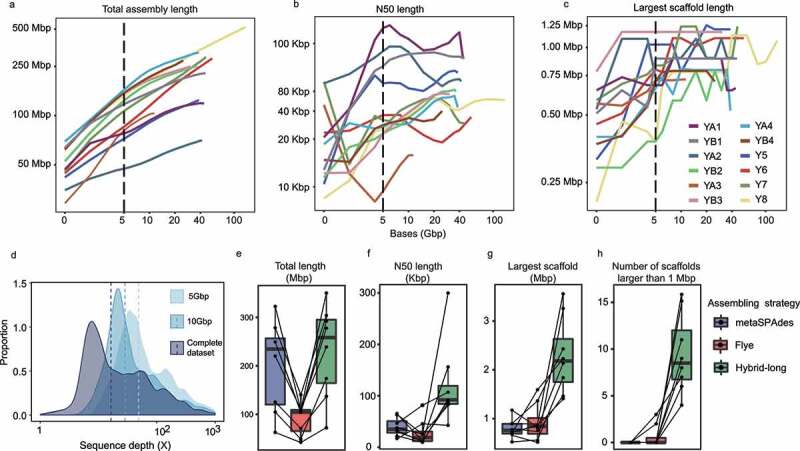
Benchmark of hybrid, ultra-deep metagenomic sequencing and metagenome assembly. Rarefaction analysis of the (a) total assembly length, (b) N50 length, and (c) length of largest scaffold against the amount of sequencing data. The total length of assembly but not other assembly performance indicators, e.g., N50 and the largest scaffold length, increased with the sequencing depth. (d) Proportion of scaffolds obtained at different sequencing depths of the complete dataset and subsampling of 5 Gbp and 10 Gbp of data. (e-h) Performance of metagenomic assembling using different strategies, including metaSPAdes (targeting short-read only), Flye (targeting long-read only), and hybrid-long (targeting both short-read and long-read). The “hybrid-long” approach produced apparently longer total assembly length (e) and higher assembly contiguity, represented by the N50 length (f) and the length of the largest scaffold (g), generating 44 scaffolds that were larger than 1 Mbp (h). Data are presented as boxplots (center line, median; box limits, upper and lower quartiles; whiskers, 1.5× interquartile range; points, outliers).

Assembly of the HiSeq data by metaSPAdes^29^ alone achieved an average N50 length of 38 Kbp, corresponding to a total of 238 Mbp of sequencing data per sample (Table S1b). Rarefaction analysis showed that, under the sequencing data amount of 5 Gbp, the total length and performance (estimated by the N50 and the largest scaffold lengths) of the assembled scaffolds increased rapidly as more sequencing data were generated (Figure 1a). The size of the assembled sequences continued to increase until the sequencing data amount reached 40 Gbp (Figure 1a), while the assembly performance measured by the N50 and the largest scaffold lengths leveled off (Figure 1b,c). Moreover, subsampling of 10 Gbp data was more effective in achieving low-abundance genomic fragments than 5 Gbp (Figure 1d), but it did not significantly improve assembly performance (Figure S1a-c). Next, the HiSeq and PacBio data of the first dataset were assembled by using an integrated hybrid plus “super-scaffolding” assembly strategy (see Methods and Supplementary Note for further details). Comparing with the results of assembly of the HiSeq data alone, the hybrid assembly improved the assembly performance by as much as 2.5 times of N50 length (mean = 2.0 ± 0.4, range = 1.2–2.5) and an additional 18.7%±18.9% of total length (range = 7.5%-67.8%), suggesting that the genome capture was greatly enhanced (Table S1b-f, Figure 1e-g). Noteworthy, >1 Mbp scaffold length was only achieved by the hybrid assembly approach, enabling the construction of 44 scaffolds of such length, but not by assembling solely the HiSeq sequencing data (Figure 1h). In addition, the hybrid assembly approach uncovered more low coverage scaffolds than genome assembling based only on the HiSeq data (Figure S1d). Our results suggested that the combined use of PacBio and HiSeq sequencing significantly improved the effectiveness in metagenomic assembly and coverage, revealing hidden genomic features and identifying low-abundance species.

### Assembling single-scaffolds of complete (circularized, no gaps) MAGs (CMAGs)

**Figure 2.**
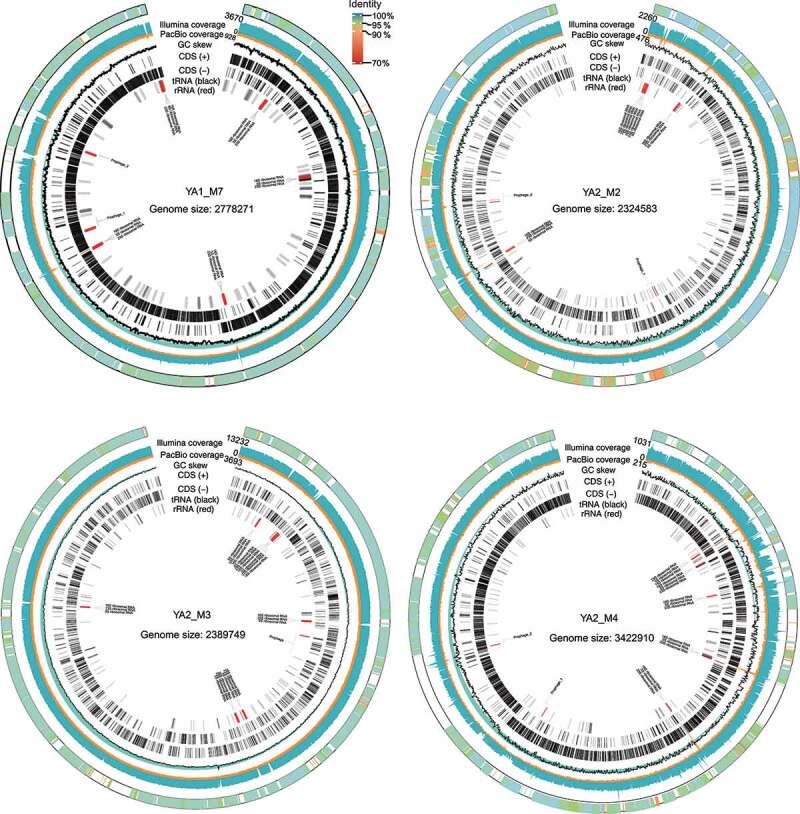
Genomic maps of four assembled complete (circularized, no gaps) MAGs (CMAGs). The CMAGs were YA1_M7, YA2_M2, YA2_M3, and YA2_M4, respectively. The CMAGs were assembled from sufficient PacBio and Illumina reads to ensure a high level of base consensus. The average level of long read (by PacBio sequencing) coverage was 757 ± 792 (range = 123–2106), and the average short read (by Illumina sequencing) coverage was 3064 ± 2960 (range = 615–8098). The outer rings represent scaffolds from the corresponding species in UHGG database that was mapped to the CMAG. The Illumina coverage, PacBio coverage, GC skew, coding sequences (CDS) of the positive (+) and negative (-) strands, and distribution of tRNA and rRNA are shown in the genomic maps.

One important achievement of the hybrid assembly approach was the successful assembly of four CMAGs.^30^ The minimum completeness of these CMAGs was 98.65%, and the maximum contamination was 1%. The assembling quality of these CMAGs was also visually verified based on the cumulative GC skew and the depth of PacBio and Illumina reads (Figure 2). The short-read depth dropped to zero at repetitive regions (e.g., regions of rRNA operons), as only unique mapped reads were considered. However, the PacBio reads could cover these locations and join the adjacent overlaps between gene fragments to correctly assemble the near-complete CMAGs in single scaffolds. Then, these CMAGs were compared with the Unified Human Gastrointestinal Genome (UHGG) dataset^14^ that contained the most comprehensive reference genomes. Four homologous UHGG species were identified (average nucleotide identity, ANI: 97.3–98.4%). One of these UHGG species was an isolated species (i.e., GUT_GENOME096210, *Faecalicatena gnavus*), which was assembled from 41 scaffolds of a metagenome dataset. The other three UHGG species were purely derived by metagenomic assembly from an average of 49 scaffolds. Thus, the CMAGs assembled in this study were the first circularized, near-complete genomes for their respective species identified in the UHGG database.

Notably, the four currently assembled CMAGs also contained the complete genome information of the corresponding bacteria, including multiple copies of rRNA operons (Table S2). In contrast, the metagenome assemblies of the four corresponding UHGG species did not have complete information of rRNA operons or genome information of prophages and repeated regions. Therefore, the current CMAG assembling method not only extended the completeness of genome assembly but also revealed genome features that were not resolved previously.

### Assembling MAGs and taxonomic assignment of microbial genomes

We further assembled the MAGs in our datasets and performed taxonomic assignment. Initially, a total of 1,781 raw bins were obtained from the scaffold set after metagenomic binning using MetaBAT2 (Table S3). Then a procedure was developed to remove the incompatible sequences, followed by merging highly similar bins, yielding 475 draft genomes that fulfilled the criteria of completeness >80%; contaminations <5%, and quality score >60. These draft genomes included over 80% Illumina reads across all samples; therefore, they were representative of the overall metagenomic contents and gut microbial communities. The draft genomes were of an average genome size of 2.9 Mbp (ranging from 1.3 to 7.4 Mbp) with an average N50 length of 92 Kbp (ranging from 5.4 Kbp to 3.7 Mbp; Table S4). Only 94 of these draft genomes satisfied the criteria of ‘high-quality genome’ according to the Minimum Information about a Metagenome-assembled Genome (MIMAG) standard^31^ (i.e. >90% completeness and <5% contamination, with 5S, 16S, and 23S rRNA genes and at least 18 tRNAs; Table S5). The majority of the high-quality MAGs (94%) were reconstructed by using the hybrid metagenomic assembling approach, and most metagenomic MAGs assembled exclusively by short-reads did not reach the “high-quality” level owing to failure to resolve the rRNA operon regions. These results suggested that the hybrid metagenomic approach could significantly improve the genome assembling quality, including the problematic regions like regions of rRNA sequences.

**Figure 3.**
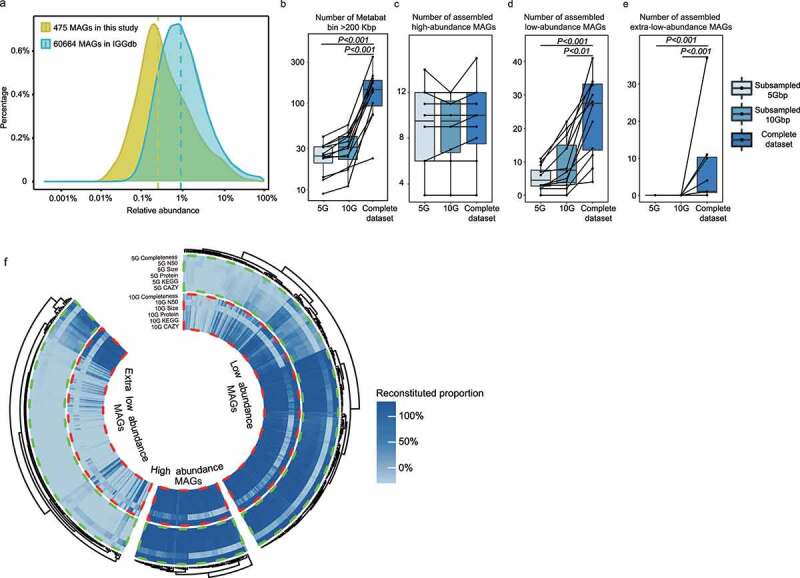
(a) Distribution of metagenomic-assembled genomes (MAGs) of different relative abundance recovered in the IGG database using routine metagenomic analysis approach and hybrid extra-deep sequencing metagenomic assembling pipeline in the current dataset. (b-e) Binning performance reflected by the number of >200 Kbp bins, high-, low-, and extra-low-abundance genomes assembled in the complete dataset, 5Gbp and 10Gbp subsampled datasets. (f) Ultra-deep sequencing outperformed shallow sequencing in assembling metagenomic-assembled genomes (MAGs). The high-, low-, and extra-low-abundance MAGs reconstructed using 5 Gbp and 10 Gbp subsampling datasets were compared with the 475-high-quality MAGs achieved by ultra-deep sequencing. The genome completeness, N50 length, genome size, number of identified coding sequences (proteins), genes identified in the Kyoto Encyclopedia of Genes and Genomes (KEGG) database, and carbohydrate-active enzymes (CAZymes) are shown. The color scale represents the reconstituted proportion of specific parameters reconstructed with the 5 Gbp and 10 Gbp subsampling datasets relative to the metagenomic assembling and annotation results achieved by ultra-deep sequencing.

Our metagenomic strategy also enabled the recovery of genomes of low-abundance species. Our results found that the inter-individual relative abundance of MAGs was approximately one order of magnitude lower than the Integrated Gut Genomes (IGG) database (Figure 3a). The IGG database was an integrated genome catalog of microbiomes of gut and other environments that comprised ~60,664 MAGs.^13^ The medium relative abundance of MAGs from common metagenomic studies was approximately 1%, which was applied as the cutoff level to distinguish between high-abundance and low-abundance taxa. Our study identified 111 high-abundance (>1% relative abundance) and 287 low-abundance MAGs (0.1–1% relative abundance). The remaining 77 MAGs each comprised <0.1% relative abundances, which were considered as extra-low-abundance species and were rarely discovered in previous metagenomic studies.

To further compare the effectiveness in uncovering microbial species at shallow sequencing depth, MAGs were reconstructed from the datasets of 5 and 10 Gbp subsamples, as 5 and 10 Gbp were the sequencing amounts used in most conventional metagenomic studies (Table S6). MetaBAT2 was initially used to assess the binning performance, and the results showed that the number of bins (of bin size >200 Kbp) generated at shallow sequencing depth was significantly lower than those produced by using ultra-deep metagenomic sequencing (Figure 3b). Then a reference base binning method was used to reconstruct the MAGs at shallow sequencing depth. Over 98% of the high-abundance MAGs were recovered with >80% completeness at 5 or 10 Gbp sequencing amount (Figure 3c). Approximately 23% of the recovered MAGs were of low-abundance, and no extra-low-abundance MAGs was recovered by sequencing 5 or 10 Gbp data (Figure 3d-e). Moreover, the gene coverage, functional genomic content, and assembly performance of low- and extra low-abundance MAGs achieved by shallow sequencing depth (5 G and 10 G) were largely insufficient compared with ultra-deep sequencing (Figure 3f, Table S7). These results suggested that ultra-deep sequencing was superior to conventional metagenomic sequencing for deciphering the low-abundance microbial subpopulation.

Taxonomic assignment of MAGs revealed seven phyla, 16 classes, 24 orders, 40 families, 72 genera, and 116 species. Most identified taxa were members of the Firmicutes (74.7%), followed by Bacteroidetes (9.5%), Actinobacteria (7.1%), and Proteobacteria (6.5%). Detected minor phyla included Desulfobacterota (four species), Fusobacteria (three species), Verrucomicrobia (two species), and Euryarchaeota (one species). Notably, nearly half of the MAGs could not be taxonomically classified to the species level (n = 234); these MAGs were regarded as uncultured species. At the phylum level, 51.0% of Firmicutes, 48.4% of Proteobacteria, and 41.2% of Actinobacteria were classed as uncultured species (Figure S2a). Among these MAGs, 90.2% of Actinobacteria, 86.1% of Firmicutes, 83.3% of Proteobacteria, and 79.5% of Bacteroidetes were species of low- or extra-low-abundance (Figure S2b).

Afterward, our MAG dataset was compared with the UHGG dataset to determine the quality and novelty of the metagenomic content in our samples. Twenty-four MAGs were identified as novel genomes with <95% ANI compared with any existing species (Figure S3; the ANI cutoff level was reference to),^32^ while 209 MAGs showed improved genomic quality compared to existing assembled genomes of the same species. Moreover, the full-length 16S rRNA genes of 167 MAGs that were missing in existing reference genomes were found in this study (Table S4). In addition, 66.7% of new MAGs found in this work were assigned to the order Clostridia. These results suggested that, despite the continuously increase in newly added genomes in recent gut microbiome studies, certain clades still contain numerous uncultured members yet to be identified and explored.

### Genomic features of species of different abundance

**Figure 4.**
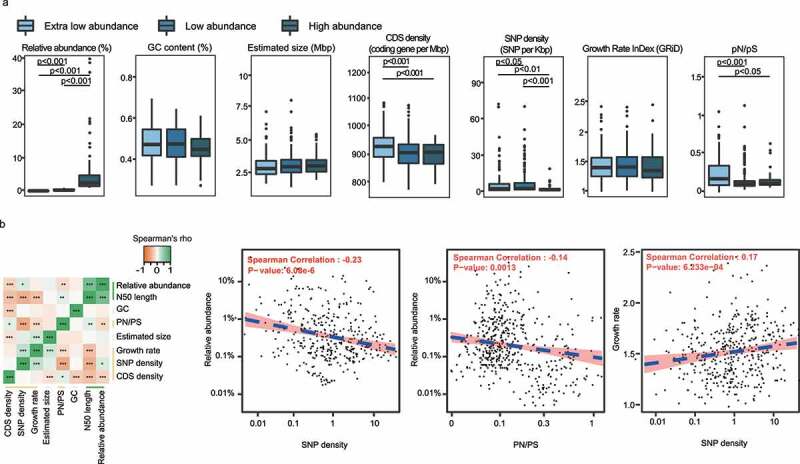
(a) Comparison of genomic features among high-, low-, and extra-low-abundance species. (b) Spearman’s correlations between different genomic features. Asterisks shown in the heatmap represent statistical significance: *p < .05, **p < .01, and ***p < .001. The blue dash line in the scatter plot indicates Spearman correlation.

The cultivability and abundance of microbes might associate with their intrinsic genomic features (i.e., GC content, estimated genome size, and density of coding sequence), growth rate, and selective pressure in the ecosystem (indicated by SNP density and pN/pS rate). Generally, low-abundance and extra-low-abundance species had higher coding density, pN/pS ratio, and SNP density than the high-abundance species (Figure 4a). Interestingly, the relative abundance correlated negatively with the SNP density (r = -0.25, *P* < .001) and weakly with pN/pS ratio (r = -0.12, *P* = .011), suggesting that there was a stronger selective pressure on the low-abundance species in the gut environment (Figure 4b). The growth rate correlated positively with the SNP density (r = 0.26, *P* < .001) and the estimated genome size (r = 0.19, *P* < .001); meanwhile, the SNP density correlated positively with the estimated genome size (r = 0.21, *P* < .001). Moreover, a notable negative correlation existed between the coding density and the estimated genome size (r = -0.18, *P* = .001; Figure 4b). The associations between these parameters might potentially reflect interspecies interactions and niche adaption amongst gut species.

### Chromosomal mobile genetic elements (MGEs)

**Figure 5.**
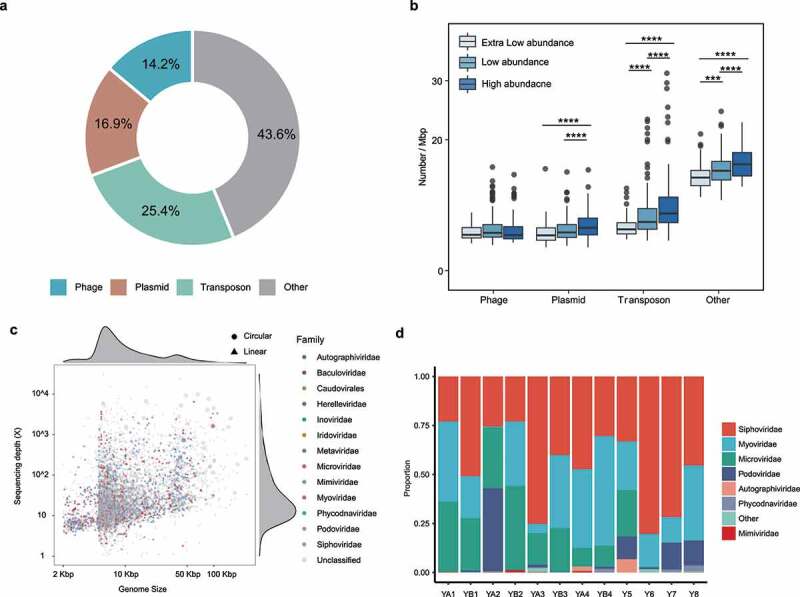
Chromosomal mobile genetic elements (MGEs) of assembled genomes. (a) Proportion of chromosomal MGEs across all metagenomic-assembled genomes (MAGs). (b) Boxplots showing distribution of MGEs across MAGs of different abundance. (c) Distribution of viral families by length and depth of sequencing coverage. (d) Family-level taxonomic composition of gut viromes in 12 individuals. ***p < .001, ****p < .0001.

The distribution of chromosomal MGEs (cMGEs) showed obvious genomic variability between species/MAGs of different abundance. A total of 38624 cMGEs were identified, including 9807 transposon-associated MGEs, 6513 plasmid-associated MGEs, 5473 phage-associated MGEs, and 16831 MGEs-associated with other mechanisms (Table S8, Figure 5a). The high-abundance species had significantly more plasmid-associated MGEs, transposon-associated MGEs, and MGEs-associated with other mechanisms than the low-abundance and extra-low-abundance species (*P < *.001 in all cases; Figure 5b).

### Extrachromosomal MGEs

Extrachromosomal MGEs were also identified. A total of 281 unbinned putative plasmids (>10 Kbp; Table S9) and 5,097 putative phages (>5 Kbp; Table S10, Figure 5c) were found in the assembled metagenomes. Three dominating viral families formed the core gut virome, including Siphoviridae (average relative abundance of 48.7%), Myoviridae (16.1%), and Podoviridae (5%) (Figure 5d, Table S10). Four single-scaffold ubiquitous human gut-associated phages of crAssphage were assembled, one of which had a circular genome of 98.0 kb. Highly homologous counterpart of the majority of identified plasmids (72%) could be identified in the NCBI database, but a large proportion of putative phages (80%) could not be classified to the family level, suggesting the existence of a large unexplored category of extrachromosomal MGEs in the human gut metagenome.

### Polysaccharide metabolism and short-chain fatty acids (SCFAs) biosynthesis-related genes in different abundant MAGs

To understand the metagenomic potential of subjects’ gut microbiomes in degrading and metabolizing common polysaccharides, the key predicted gut metabolic pathways and interactions of MAGs networks in each individual were reconstructed via annotation based on key reactions in the Kyoto Encyclopedia of Genes and Genomes (KEGG) database. Pathways were detected using Omixer-RPM (v.1.0) described by Vieira-Silva et al.^33^ The biodegradation of nondigestible starch particles, plant and host-derived polysaccharides (summarized as C1-6) were likely the main energy and carbon sources for the gut-degrading microbes. Subsequently, organic acids (including lactate and succinate) and SCFAs were likely produced after sugar fermentation by the gut anaerobes.

**Figure 6.**
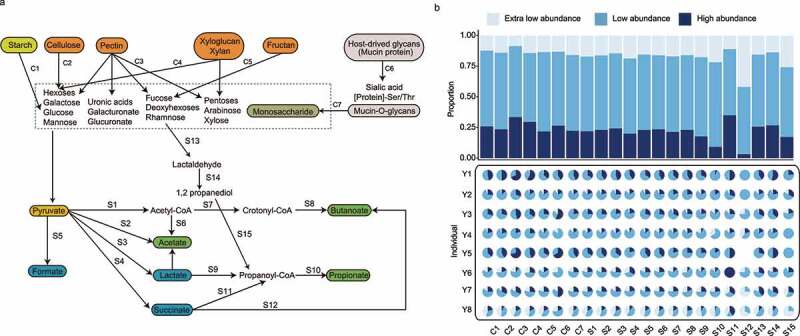
Predicted metabolic potential of gut metagenomes. (a) Schematic diagram illustrating polysaccharide metabolism and short-chain fatty acid (SCFA) biosynthesis-related metagenomic-assembled genomes (MAGs) identified in our datasets. The polysaccharide metabolism and SCFA biosynthesis-related pathways are represented by the codes C1-C7 and S1-S15, respectively. Functional gene annotation was performed based on key reactions in the Kyoto Encyclopedia of Genes and Genomes (KEGG) database, and pathways were detected using Omixer-RPM (v.1.0) described by Vieira-Silva et al. The detail of each module reaction is shown in Table S12. (b) The stacked bar chart shows the overall distribution of the relevant pathways (C1-C7, S1-S15) across the high-abundance, low-abundance, and extra-low-abundance MAGs. The pie charts show the breakdown of these pathways in each individual.

Taxa represented by the extra-low abundance and low-abundance MAGs seemed to be major players participating in most metabolism-related pathways, accounting for an average of 16.3% and 58.3% of such metabolic functions (Figure 6). In particular, nine pathways were enriched among the low-abundance species (Table S11), including some polysaccharide-degradation pathways including starch degradation (C1), cellulose degradation (C2), xyloglucan and xylan degradation (C4), and fructan degradation and some organic acids and SCFA synthesis pathways including lactate (S3) and propionate (S10). These results suggested that the low-abundance species might be active participants in the gut polysaccharide metabolism and SCFAs biosynthesis.

## Discussion

Most metagenomic studies have limited data acquisition to 5–10 Gbp per sample; however, our results revealed that novel genomic features would still be obtained with a sequencing depth beyond 10 Gbp of short-read sequencing. When the sequencing depth further increased, significantly more low-abundance species would be uncovered, suggesting that conventional metagenomic sequencing and assembly approach would miss a large portion of microbial biodiversity and many of the low-abundance species that are yet to be discovered and explored. The limited efficiency of assembling short-read metagenomes^34^ could be overcome by combining long-read sequencing.^22,35^ Thus, this study employed a hybrid assembly strategy to connect long-read and short-read contigs.

Our strategy was advantageous in ensuring the correctness of assembling repeated regions and generating contiguous gapless assemblies. This strategy boosted assembly performance, enabling the achievement of a much more comprehensive overview of the microbiome composition and thus deeper understanding of complex microbial communities. In particular, our hybrid assembly pipeline successfully assembled high-quality single-scaffold genomes, enabling us to obtain high-quality reference genomes directly from complex metagenomic samples. Our strategy was also effective in attaining high-quality and complete single-scaffold bacterial genome spanning multiple rRNA operons, which has not always been achievable in previous works. For example, our work successfully assembled four high-quality CMAGs that were the first representatives of their assigned species. These four genomes were assembled to gapless single-circularized genomes that contained complete rRNA operons, including the 5S, 16S, and 23S rRNA genes. In contrast, their counterpart reference genomes were fragmented multiple contigs, missing one or more of the 5S/16S/23S gene features even if rRNA operon-like sequences were identified. Thus, the availability of these representative genome sequences largely improved the accuracy of taxonomic annotation, genomic analysis, and 16S rRNA gene-based abundance analysis as a result of eliminating the factor of copy number variations.

The hybrid assembly of ultra-deep metagenomic sequencing largely improved the performance of metagenomic analysis, particularly in the aspect of data extraction and assembly. By using such pipeline, this study assembled 475 draft genomes from 12 human feces samples. Twenty-four novel genomes were assembled, and the assemblies of 47 existing genomes were significantly improved. The MIMAG standards were proposed by the Genomic Standards Consortium for reporting bacterial and archaeal genome sequences. The standards have defined the criteria of high-quality draft MAGs based on genome completeness (>90% completeness; including the 23S, 16S, and 5S rRNA genes, and at least 18 tRNAs) and contamination (<5% contamination). Approximately 20% of the draft genomes assembled in this study could be classified as high-quality according to the definition laid out in the MIMAG standards, and most of them (94%) were assembled by the hybrid approach, indicating that the currently developed genome assembling pipeline was a lot more effective in achieving high-quality genomes. A recent study compiled and analyzed 204,938 genomes from human gut microbiome datasets published previously in short read-only MAGs studies, and clustering analysis revealed a total of 4,644 prokaryotic species. Among them, only 573 (12.3%) representative genomes fulfilled the high-quality criteria laid out in the MIMAG standards.^14^ Our findings implicated that a substantial degree of bacterial diversity remained unexplored and that the quality of existing genomes should be raised, even though hundreds of thousands of MAGs had been reconstructed from tens of thousands of metagenomic samples.

The continuing increase in sequencing throughput greatly facilitated the identification of low-abundance species. By adopting an assembly pipeline based on long-read sequencing, the detection threshold for low-abundance species decreased to 0.1%; and such level of detection sensitivity was an order of magnitude lower than that achieved in conventional gut metagenomic studies. Walsh et al. (2018) showed that the metagenomic sequencing depth would not significantly affect the outcome of taxonomic and/or functional analysis of low-diversity microbiomes.^36^ Our study however demonstrated that the sequencing depth significantly improved the metagenomic binning performance of low-abundance species in complex human gut microbiomes, especially species below 1% relative abundance. Our findings suggested that the genomes of the majority of low-abundance and extra-low-abundance species could not be reconstructed readily by applying metagenomic sequencing of shallow sequencing depth.

Our strategy would also reveal the ecological niche of the sampling environment, as the habitat niche is recognized as the major force shaping the microbiota composition and relative abundances of individual bacterial species.^37^ Our study found that there were fewer MGEs among the low-abundance MAGs compared with the highly abundant ones. It was unlikely that the smaller number of MGEs present among the low-abundance MAGs was due to the effect of genome incompleteness, as our analysis procedure did normalize the number of cMGEs by the genome size to eliminate such effect. It was also unlikely that the lower number of plasmid-associated MGEs detected among the low-abundance MAGs was simply due to biases created by the unsymmetrical number of reads between the high-abundance and low-abundance MAGs in the metagenomic dataset, as some functional genes/pathways were indeed detected to be more abundant among the low-abundance MAGs than the high-abundance ones in some individuals, e.g., those involving in microbial degradation of polymerized carbohydrates. On the other hand, the present study found that the low-abundance species had a higher SNP density compared with the high-abundance species, supporting that the low-abundance species were under stronger selection pressure than the high-abundance species. The presence of MGEs has also been suggested to be related to the evolvability and fitness of the gut microbiome.^38–40^ Yet, the current data should be interpreted carefully due to the limitation of a small sample size in this study and the unsymmetrical number of reads between the high- and low-abundance MAGs in the dataset. Both the number of samples and depth of sequencing would have to be increased in future studies to consolidate the current findings. Nevertheless, our observations make studying these lowly abundant gut microbes crucial to better understand the gut ecology.

The genomes of low-abundance species from some individuals contained more genes coding pathways involved in microbial degradation of polymerized carbohydrates, and their fermentative products were upstream metabolites of SCFAs and other organic acids. These results suggested that this low-abundance subpopulation might contribute greatly to various colonic microbial metabolism and fermentation, which are crucial and are beneficial to the host.^41–43^ Thus, it would be necessary to describe and characterize these previously under-recorded species at the individual level, so as to understand their activities in the gut environment and their contributions to the host. This is so far only possible by using currently developed hybrid deep metagenomic sequencing and genome assembly pipeline.

Long-read metagenomic sequencing has been shown to be an effective approach for identifying extrachromosomal MGEs in human gut microbiome.^26^ It has been reported that MGEs play important roles in microbial evolution and adaptation, and they are also involved in host interactions.^44,45^ Our pipeline identified dozens of circular plasmids and thousands of phages, most of which had no homology to known species. Thus, the annotation and understanding of human gut extrachromosomal MGEs found in this study were hampered by the lack of reference genomes. On the other hand, this represents a great opportunity for use of the method developed here to describe and uncover novel MGEs in human gut microbiomes.

The hybrid assembly of ultra-deep metagenomic sequencing pipeline developed in this work could reveal more complete information on the functional metagenomic level, highlighting the value of deep sequencing in unveiling thorough genomic features and functional metagenomic potentials of rare species existing in complex microbiota. Nevertheless, one big disadvantage of this approach is the high cost, up to approximately 15–20 times more expensive than shallow short-read sequencing. Thus, to obtain a comprehensive metagenomic snapshot including the rare species in complex microbial communities, one alternative way would be to sequence a relatively low but representative number of sample cohorts instead of applying merely low-coverage sequencing, which has been adopted by most conventional metagenomic studies.

## Conclusion

The continuous increase in sequencing throughput has generated an enormous amount of metagenomic data. However, conventional metagenomic sequencing approaches usually employ a relatively shallow sequencing depth, which limits the detection sensitivity of the low-abundance and extra-low-abundance subpopulations in complex microbial communities. By developing an ultra-deep, hybrid metagenomic assembly pipeline, the current work successfully reconstructed high-quality gapless genomes of some low-abundance and extra-low abundance species from human gut metagenomes. Our results confirmed that these minor microbial subpopulations carried novel and specific genomic features, particularly patterns of MGEs and metabolic pathways, suggesting that they might play specific role within the gut microbial community and contribute actively to the host. Although the current pipeline significantly enhanced the binning performance, improved the quality of reference genome dataset, and captured low-abundance species, gaining a thorough understanding of the gut microbial communities and their interactions at the individual level remains challenging and costly.

## Methods

### Metagenomic samples and dataset

Eight fecal samples were obtained from four individuals (two samples from each individual collected seven days apart), and four other fecal samples were donated by another four individuals. The HMP mock community dataset (https://github.com/PacificBiosciences/DevNet/wiki/Human_Microbiome_Project_MockB_Shotgun) was retrieved to optimize the metagenome assembly workflow.

### DNA extraction for metagenomic samples

Metagenomic DNA was extracted from feces using a MagaZorb DNA Mini-Prep Kit (batch: MB1004), in accordance with the manufacturer’s instructions. The quality of the extracted DNA was checked using agarose gel electrophoresis on a 0.6% (w/v) agarose gel, and the quantity of DNA was determined using a Qubit2.0 fluorometer.

### Preparation of SMRTbell library and SMRT sequencing

Library construction and SMRT sequencing were performed following standard recommended protocols (Procedure and Checklist -20 kb Template Preparation, Pacific Biosciences of California, Inc., USA). Indeed, we experienced technical problems in constructing a 20Kb library for long-read SMRT metagenomic sequencing, as some DNA fragments were damaged during the extraction and purification procedures. To get high molecular weight and high-quality metagenomic DNA fragments for library preparation, the damaged DNA was repaired before reattempting library preparation using the New England BioLabs PreCR® Repair Mix Kit according to the manufacturer’s instructions. This step was crucial to library preparation. Subsequently, a large insert library was constructed for each sample using the SMRTbell™ Template Prep Kit, following the PacBio’s instructions for 20 kb template preparation.^45^ After that, the Binding Calculator (version 2.3; Pacific Biosciences of California, Inc., USA) was used to calculate the binding/annealing reactions and the concentration of bound complex to be loaded onto the sample plate for the instrument. Before sequencing, the size-selected SMRTbells were annealed with suitable primers. Next, the annealed libraries were bound to the P6-C4 enzyme using a ratio of 10:1 polymerase to SMRTbell. The SMRTbell library sequencing was done by PacBio RS II (Pacific Biosciences of California, Inc., USA) on eight SMRTcells after a magnetic bead-loading step specified in the manufacturer’s instructions.

### Illumina library preparation and sequencing

DNA libraries were prepared by using the NEBnext® Ultra™ II DNA Library Prep Kit for Illumina® (New England BioLabs). The Illumina HiSeq X Ten platform was then used for 2 × 150 bp paired-end whole-metagenome sequencing with a target sequencing depth of at least 20 Gbp raw data per sample.

### Hybrid metagenomic assembly

KneadData v0.7.5 (http://huttenhower.sph.harvard.edu/kneaddata) was used to remove the low-quality and human genome sequences for short-read sequencing data. The long reads were mapped to the human reference genome (GRCh38) using minimap2^46^ (“-x asm5”) to remove human genome sequences. An integrated hybrid metagenomic assembly methodology was employed to construct “super scaffolds”. An overview is shown in Figure S4. Firstly, the long reads were used to construct most contigs, while the short reads were used to polish the long-read contigs and supplement sequences missing in the long reads. The long reads were assembled using Flye^47^ (version: 2.8) with the parameters ‘–meta’ and ‘–pacbio-raw’. Two rounds of Racon (v1.4.10, link https://github.com/lbcb-sci/racon) were then applied to the layouts to obtain the consensus sequences. Two rounds of Pilon^48^ polishing (v1.23, round 1: “–fix all,amb,circles”, round 2: “–fix all”) were applied to the consensus sequences utilizing the short reads. However, we found that the long reads assemblers failed to assemble the low-abundance genomes in the mock community dataset efficiently (Supplementary Note). To address this issue, the HybridSPAdes^17^ was used to assemble both short and long reads, and two separate assemblies derived from HybridSPAdes and Flye were used in combination with Quickmerge.^49^

### Illumina metagenomic assembly

High-quality Illumina metagenomic samples were assembled by metaSPAdes (3.13.0),^29^ with the parameters -k 33,55,77,99,111 -meta. Scaffolds with lengths <2,000 bp in each assembly were removed before metagenomic assembly evaluation and binning. QUAST (version: 5.0.0) was used to evaluate the resulting metagenomic assemblies with “–min-contig 2000”.

### Genomes reconstructed from metagenomes

Illumina-scaffold or super-scaffold binning was done based on tetranucleotide frequency and scaffold abundance using MetaBAT2.^50^ However, MetaBAT2 often failed to reconstruct genomes and generated multi-bins of the same microbial population that were of low quality and completeness. To avoid this, a method which clustered scaffolds (based on sequence features, coverage, and homology) was used, and this method was guided by the presence of marker genes (Figure S2b). Briefly, the characteristics of each scaffold were prepared, including taxonomy, coverage, GC content, tetranucleotide frequency (TNF), and single-copy gene (SCG) information. The taxonomy of each scaffold was assigned and annotated by searching against the NCBI nonredundant Nucleotide Sequence Database (NT) and Kraken with default parameters.^51^ The genes of the scaffolds used for downstream analysis were then predicted using Prodigal (v2.6.3)^52^ with the meta option. The predicted genes within scaffolds were searched against the UniProt TrEMBL database (UT) using Diamond, and the SCG information of the scaffolds was determined using a custom SCG set of 123 SCGs chosen from the Pfam database (Version 31) using HMMER.^53^ The custom SCG set was constructed by filtering out SGCs that were not conserved across most bacteria (>3000 bacteria species in NCBI database) based on a previously reported list of universal SCGs for bacteria.^54^ The method for assessing taxonomically unassigned MAGs was adopted from the study of Stewart et al.^55^ After preparing the scaffold information, an iterative and score-based procedure was used to generate the clusters. The python scripts for these binning methods were made available under the web address, https://github.com/jinhao94/hybrid_script.git.

### Estimating the abundance of MAGs

BWA MEM (v.0.7.17)^56^ was used to map reads to the scaffolds; and samtools (v.1.9)^57^ was used to convert the output file to BAM format. The average depth for each scaffold in each MAG was calculated using MetaBAT2 script jgi_summarize_bam_contig_depths. The depth for each MAG was calculated by the average of each scaffold in the MAG and normalized by scaffold length. The relative abundance of each MAG was computed as the depth of the MAGs normalized by the total reads of the metagenome sample to allow for sample-to-sample comparison. Long reads were aligned to CMAGs using Minimap2 (version: 2.16-r922),^46^ excluding secondary alignments using samtools. The nanopore coverage was calculated using bedtools genomecov (version: 2.27.1).^58^ Average per-window depth was computed using mosdepth (version: 0.2.5)^59^ with a window size of 1000 bp and visualized using the circos package in R.

### Genome quality and comparative genomics

The completeness and contamination of each of the recovered genomes were estimated using CheckM (v1.0.18)^60^ lineage-workflows with default parameters. 16S rRNA genes were predicted using barrnap (v.0.9, https://github.com/tseemann/barrnap). The estimated genome size was adjusted to account for its completeness and contamination: *Estimated genome size = (genome size)/(completeness + contamination)*. The genome replication rate was calculated using the GRiD software (version 1.3).^61^ This method calculated the genome growth rate from reference genomes at ultra-low sequencing coverage (>0.2x) based on estimating the ratio between coverage at the peak (ori) and the terminus (ter) for the reference bacterial genome using redescending M estimator with Tukey’s biweight function. The GRiD value was directly proportional to the growth rate.

### Identification of novel species

High-quality MAGs sequences were compared to the species in the UHGG dataset using fastANI, with coverage of at least 40% of the MAG and at least 95% ANI. Then, the new MAGs were clustered at the species level using dRep v2.2.4 with the following parameters ‘-pa 0.9 -sa 0.95 -nc 0.30 -cm larger’. To identity the genome quality improvement for the existing reference genomes, the score for each genome was calculated using the following formula: *Score = completeness – 5´ contamination*. The genome in our dataset having a higher score than the corresponding genome in the UHGG dataset was regarded as “quality improved genome”.

### Estimation of SNP density and pN/pS

To detect SNPs in each MAG, paired-end reads of each sample were mapped to MAGs using the bwa mem algorithm, and reads with low mapping and sequence quality were discarded (quality scores <20 and <30, respectively). To avoid the influence of different depths, 40 mapped reads per site were subsampled for each MAG; MAGs without 200,000 sites of ≥20 × depth were excluded from further analysis. Bcftools mpileup was used for SNP calling, and positions with major allele frequencies of <95% with at least two matching reads of particular alleles were retained for analysis. The SNP density was calculated as the number of SNPs per kilobase. The method for calculating the natural selection (pN/pS) ratio was adopted from Schloissnig et al (2012),^62^ and the in-house script used for performing this analysis was made available under https://github.com/jinhao94/PNPS.

### Genome function analysis

For each MAG, ORFs were predicted using Prodigal (version 2.6.3) with default parameters. Several methods were employed for functional annotation. The ORFs were annotated with the KEGG database (as of 2017) using usearch (v.11.0.667_i86linux32)^63^ with -usearch_local -id 0.3 -query_cov 0.7 options, and against the CARD (download at 2019.8) database^64^ using Diamond (v.0.9.25)^65^ with identity ≥95% and coverage value ≥0.9. Functional enrichment analysis was done using a one-sided Fisher’s exact test with the P value adjusted by the Hochberg method in R (v. 3.5.2). Identification of polysaccharide metabolism and SCFA biosynthesis pathways were based on key reactions in the KEGG database, and pathways analyzed in this study were shown in Table S11. Pathways were detected using Omixer-RPM (v.1.0) described by Vieira-Silva et al.^33^ The presence of pathway was defined as identification of >66% of key reactions in an MAG. The CAZymes were annotated using dbCAN2 with ‘diamond’ mode.^66^

### Identification of chromosomal MGEs

To annotate the MGEs, the open reading frames (ORFs) of each MAG were queried using Diamond blastp against the nr database (e-value <1e-10), and the best hits were then submitted to a keyword search for gene descriptions depicted in Brito et al.^38^

### Detection of extrachromosomal MGEs

Putative viral sequences were identified by integrating the search results against viral protein reference databases, including the viral signal detected tools (VirSorter)^67^ and the virus k-mer signatures model tools (VirFinder).^68^ Scaffolds ≥5 kb were assigned to VirSorter categories 1–2 or 4; VirFinder score of ≥0.9 and p < .01 was extracted for further analysis. Taxonomic annotation of viral scaffolds compared viral scaffold proteins against the Viral RefSeq using Diamond blastp with a majority-rules approach. It was considered part of that viral taxonomic group if over 50% of proteins were assigned to the same family using Diamond with a bitscore >50.

## Supplementary Material

Supplemental Material

## Data Availability

All sequencing data generated in this study can be found under NCBI BioProject PRJNA602101 (Illumina and PacBio). The in-house scripts for performing bioinformatics analyses in this work can be found under https://github.com/jinhao94/hybrid_script.git. The high-quality MAG sequences recovered in this study are available under NCBI BioSample accession numbers SAMN23526282 to SAMN23526756, as well as through https://www.dropbox.com/sh/7ixbbo4qitt12yw/AADbU33evBtohVigrPvgT-Csa?dl=0).
