# Supplementary figures and images for: Hybrid, ultra-deep metagenomic sequencing enables genomic and functional characterization of low-abundance species in the human gut microbiome
Source: Gut Microbes. 2022 Jan 22;14(1):2021790. doi: 10.1080/19490976.2021.2021790 (PMC8786330; doi:10.1080/19490976.2021.2021790)

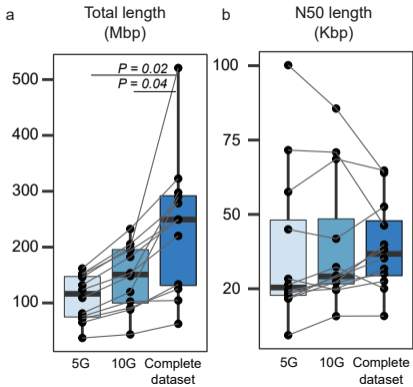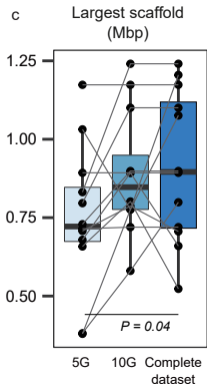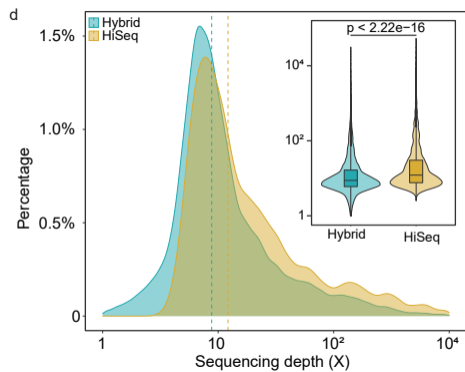

Supplement: Supplemental Material [file KGMI_A_2021790_SM0629.zip › supplementary/Figure S1.pdf]

a

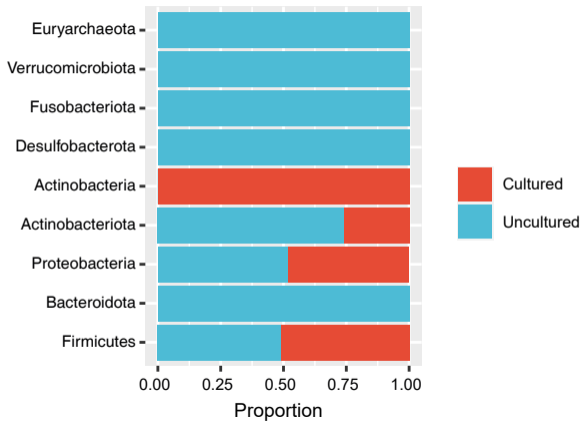

b

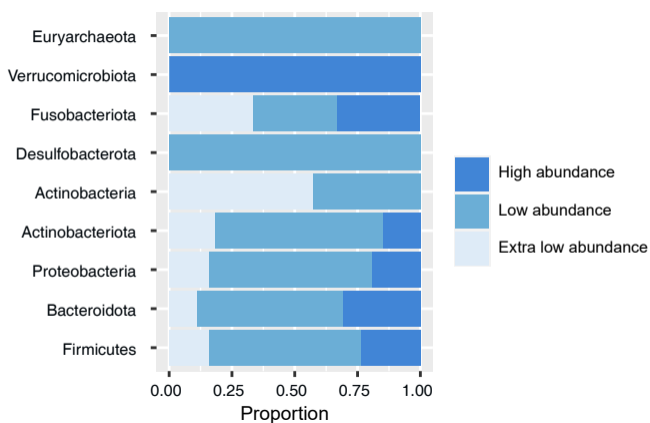

Supplement: Supplemental Material [file KGMI_A_2021790_SM0629.zip › supplementary/Figure S2.pdf]

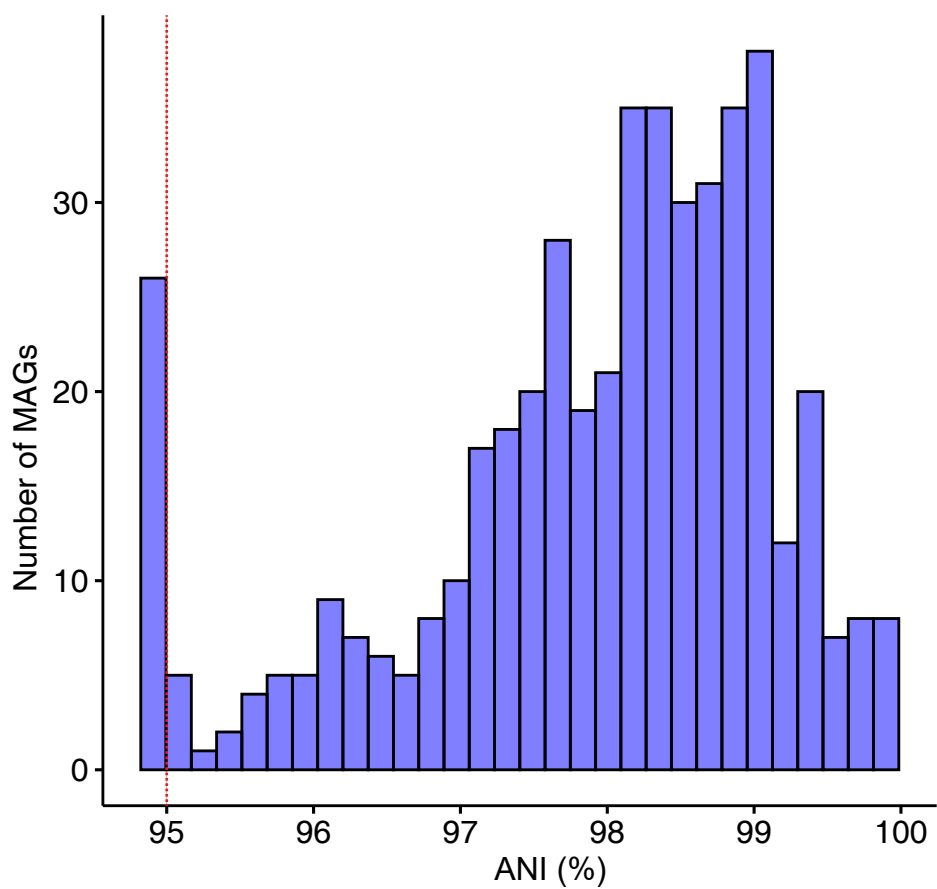

Supplement: Supplemental Material [file KGMI_A_2021790_SM0629.zip › supplementary/Figure S3.pdf]

(a)  
Assembly

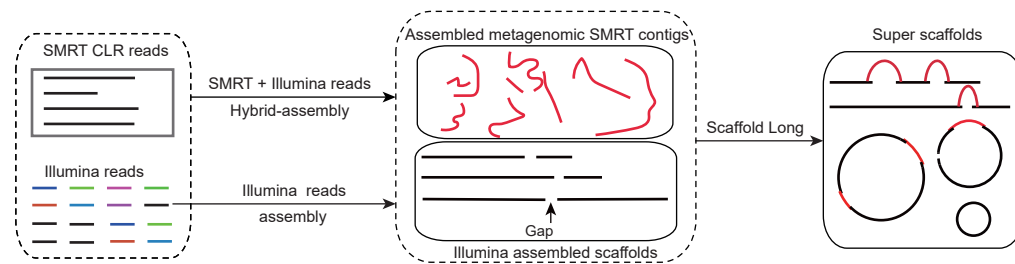

(b)  
Binning

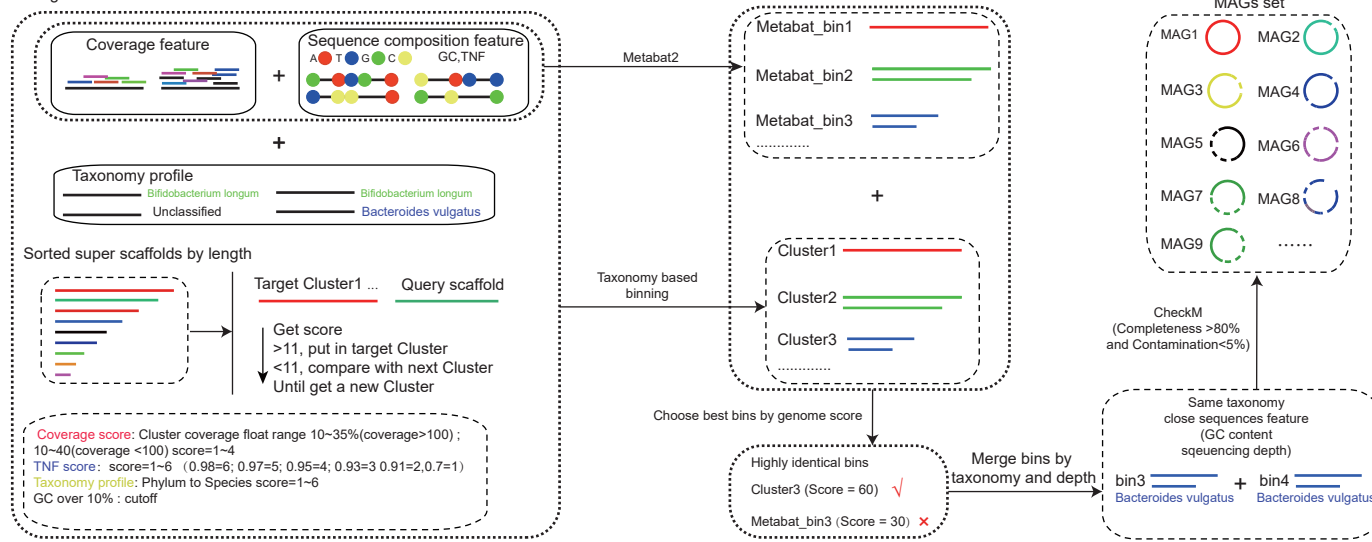

Supplement: Supplemental Material [file KGMI_A_2021790_SM0629.zip › supplementary/Figure S4.pdf]
