# Supplementary material for: Hybrid, ultra-deep metagenomic sequencing enables genomic and functional characterization of low-abundance species in the human gut microbiome
Source: Gut Microbes. 2022 Jan 22;14(1):2021790. doi: 10.1080/19490976.2021.2021790 (PMC8786330; doi:10.1080/19490976.2021.2021790)
Supplement: Supplemental Material [file KGMI_A_2021790_SM0629.zip › supplementary/supp note.doc]

Supplementary Note: **Evaluation of** **hybrid metagenomic sequencing assembly pipeline using the Human Microbiome Project (HMP) mock dataset**

To design a suitable assembling pipeline for PacBio metagenomic samples, the assembly methods for analyzing complex communities were evaluated using the HMP mock community dataset (The common genome assemblers for short-read, long-read, and hybrid read were evaluated for their accuracy and performance. The results showed that the long-read assemblers (i.e., Flye, Miniasm) had 6-7-fold contiguity (NGA50) improvement compared with short-read assemblers (i.e., MEGAHIT, metaSPAdes), and the most obvious improvement was shown when the hybrid assembler was used (7.6-fold for OPERA-MS and 1.3-fold for hybridSPAdes). These differences were also apparent in improving assembly error rate (evaluated by mismatches and misassembles). MEGAHIT and Flye (polished) were the most accurate short-read and long-read assemblers, respectively. However, long read assemblers failed to assemble the low-abundance genome efficiently, as the genome NC_003028.3 *Propionibacterium acnes* KPA171202 was either not detected or only found in low proportion compared with the results generated by short-read assemblers. To optimize the assembly performance, two separate assemblers (hybridSPAdes and Flye) were used in combination, and the subsequent results were combined with Quickmerge. After assembly merge, the efficiency of assembling of low-abundance genomes improved significantly (e.g., the assembly of NC_003028.3 increased from 0 to 97.9%, while the assembly of NC_007493.2 increased from 80.9 to 99.9%). Moreover, the assembly contiguity (NGA50) of all genomes improved slightly after assembly merge, and the assembly accuracy was not significantly affected (*P >* 0.05).

Metagenomic binning is the process of grouping assemblies of the same genome for further analysis. Our binning results were evaluated by using a reference-based binning approach, which revealed substantial differences in binning performance. The mean genome completeness calculated by Flye-hybridSPAdes was 99.2% (one genome was unbinned due to low genome fragment rate), which was higher than the results obtained by MEGAHIT (i.e., 98.7% with hybridSPAdes; 93.5% with Flye). The contamination rate calculated by Flye-hybridSPAdes was 0.7%, which was slightly higher than the results generated by Megahit (i.e., 0.2% versus an average of 0.7% when all methods were used). The assembly completeness was calculated from the difference between values generated by the short- and long-read assemblers, considering the different sequencing depths between the two methods (19.8 Gbp for Illumina versus 6.8 Gbp for PacBio). In this case, the short-read revealed more genomic details, while the long-read enabled high contiguity assembly. Overall, the genomes assembled by sequences generated singly by the PacBio or the Illumina platform were of lower contiguity and completeness compared with those generated after merging.
